# The Microbiome and Metabolic Dysfunction-Associated Steatotic Liver Disease

**DOI:** 10.3390/ijms26072882

**Published:** 2025-03-22

**Authors:** Diren Beyoğlu, Jeffrey R. Idle

**Affiliations:** 1 Department of Pharmaceutical and Administrative Sciences, College of Pharmacy and Health Sciences, Western New England University, Springfield, MA 01119, USA; diren.beyoglu@wne.edu; 2 Department of Biomedical Research, University of Bern, 3008 Bern, Switzerland

**Keywords:** MASLD, microbiota, dysbiosis, bacteriophages, bile acids, tryptophan, histidine, probiotics, intermittent fasting, phage therapy, holins, endolysins

## Abstract

Metabolic dysfunction-associated steatotic liver disease (MASLD) is a condition wherein excessive fat accumulates in the liver, leading to inflammation and potential liver damage. In this narrative review, we evaluate the tissue microbiota, how they arise and their constituent microbes, and the role of the intestinal and hepatic microbiota in MASLD. The history of bacteriophages (phages) and their occurrence in the microbiota, their part in the potential causation of MASLD, and conversely, “phage therapy” for antibiotic resistance, obesity, and MASLD, are all described. The microbiota metabolism of bile acids and dietary tryptophan and histidine is defined, together with the impacts of their individual metabolites on MASLD pathogenesis. Both periodontitis and intestinal microbiota dysbiosis may cause MASLD, and how individual microorganisms and their metabolites are involved in these processes is discussed. Novel treatment opportunities for MASLD involving the microbiota exist and include fecal microbiota transplantation, probiotics, prebiotics, synbiotics, tryptophan dietary supplements, intermittent fasting, and phages or their holins and endolysins. Although FDA is yet to approve phage therapy in clinical use, there are multiple FDA-approved clinical trials, and this may represent a new horizon for the future treatment of MASLD.

## 1. Introduction

Steatotic liver disease has recently been adopted as an all-embracing term for the various etiologies of steatosis. The new nomenclature of metabolic dysfunction-associated steatotic liver disease (MASLD) is intended to replace nonalcoholic fatty liver disease (NAFLD), alcohol-related liver disease (ALD) and nonalcoholic steatohepatitis (NASH). We have discussed the transitions in nomenclature from NAFLD to MAFLD and finally to MASLD, and how these came about [1]. MASLD is a sex-dimorphic disease. It has a higher prevalence in men than in women of reproductive age. After menopause, this sexual dimorphism disappears [2]

The gut microbiota comprises an assembly of microorganisms belonging to different kingdoms, including bacteria, archaea, fungi, and viruses, that inhabit the gastrointestinal tract of animals microorganisms [3]. The microbiome is defined as the totality of the genetic makeup of bacteria in the body [3,4]. Fifteen years ago, the gut microbiota or its products were suspected of contributing to the pathophysiology of MASLD, in particular, the proinflammatory and metabolic effects of lipopolysaccharide (LPS) arising from Gram-negative bacteria in the gut [5]. Most evidence at that time arose from animal models, but research on human MASLD was beginning to gather pace, especially as the human gut microbiome had just been sequenced and reported [6]. It soon became apparent that the gut microbiota affected host metabolism by improving energy yield from food and modulating dietary or host-derived metabolites that modify host metabolic pathways [7]. Knowledge about the contribution of the gut microbiota to human physiology and pathology started to accumulate 10 to 15 years ago [7,8,9,10]. At that time, understanding the part played by the gut microbiota in obesity, and one of its major consequences, MASLD [1], was seen as presenting a unique set of problems, in particular regarding the technical limitations of microbial identification [10] and metabolite identification. These problems were to be largely solved by the subsequent development of next-generation sequencing (NGS) and ultra-performance liquid chromatography–quadrupole time-of-flight mass spectrometry (UPLC-QTOFMS) [11]. The pros and cons of applying omics technologies in the evaluation of microbiomes have been discussed by several authors [4,12,13].

MASLD is characterized by fat accumulation, accounting for a minimum of 5% of hepatic mass, a condition referred to as steatosis. This deduction is ascertained through the biopsy/histology or imaging of the liver. MASLD can progress to the more aggressive metabolic dysfunction-associated steatohepatitis (MASH), which is characterized by the presence of hepatic steatosis surpassing 5%, accompanied by indications of hepatocellular injury, such as hepatocyte ballooning, inflammation, and advanced fibrosis [1]. MASH may subsequently progress to cirrhosis, which increases the risk of hepatocellular carcinoma (HCC) by about 2% per annum [14]. Liver transplantation is a viable option for patients with MASH and HCC [15,16], with a post-transplant survival of 88% and an average follow-up of 2.5 years [15]. A meta-analysis of 25 studies showed that patients with MASH-associated HCC were largely obese (75%) and diabetic (59.8%) [16], and these were major risk factors for MASLD that may be both genetic or diet-related in origin [17]. Moreover, evidence began to accumulate 15 years ago that the gut microbiota were involved in the development of obesity and metabolic syndrome, playing a potential role in the pathogenesis of MASLD [5,18]. As we have previously discussed [11], five phyla mainly comprise the organisms of the human intestinal microbiota, although these proportions vary from study to study. In one report [19], the proportions were Firmicutes (79%—*Ruminococcus*, *Clostridium*, *Eubacteria*), Bacterioidetes (17%—*Porphyromonas*, *Prevotella*), Proteobacteria (1%), Actinobacteria (*Bifidobacterium*) (2.5%) and Verrumicrobia (0.1%). Another estimate used nine million unique gut bacterial genes [20] from 13 bacterial phyla, with Firmicutes and Bacteroidetes comprising 64.9% and 30.4%, respectively, with the remaining 11 phyla occupying 4.7% (Proteobacteria, 1.6%; Verrucomicrobia, 1.5%; Actinobacteria, 1.4%; others, 0.2%). This topic of the intestinal microbiota as it relates to MASLD will be returned to later.

## 2. The Tissue Microbiota

### 2.1. General Considerations

For many years, it was believed that symbiotic microorganisms inhabited only the gastrointestinal tract, the mouth, and the skin. However, the presence of bacteria has been reported at many other sites. One early study found *Escherichia coli* in the urinary tracts of mice up to 2 months following inoculation with uropathogenic *E. coli*, and subsequent PCR analysis of a plasmid reporter gene (*gfp*) specific to the rifampicin-resistant *E. coli* employed produced a clear positive signal for bacterial DNA isolated from mouse bladder homogenates. The authors described these as viable but not culturable (VBNC) bacteria [21], representing a bacterial survival strategy that had been reported some time earlier [22,23]. This work was extended to include human urine specimens from women without a urinary tract infection. The samples were found to contain considerably more viable than culturable bacteria [24]. These VBNC bacteria therefore occur in regions of the urinary tract previously considered sterile. VBNC bacteria have been detected in drinking water [25], and therefore it is likely that humans and laboratory animals are continuously exposed to these organisms. There has been much discussion with regard to the dormant states of these vegetative, non-sporulating bacteria [26]. One particular concern was the continuous release of the endotoxin lipopolysaccharide (LPS) from dormant reservoirs of Gram-negative bacteria. LPS is a well-known inflammatory agent that would normally be cleared [27]. As we have recently discussed, LPS is a component of the outer membrane of Gram-negative bacteria, where it occupies up to 80% of the bacterial surface in *E. coli* and *Salmonella*, and from where it can be secreted in outer membrane vesicles [1,28]. Passage from the microbiota across the gut permits LPS to interact with the toll-like receptor 4 (TLR4), predominantly on monocytes, macrophages, and dendritic cells, leading to the activation of innate immunity with the release of proinflammatory cytokines and chemokines [1,29]. TLR4 is expressed in all parenchymal and nonparenchymal cells of the liver, whereby its activation by LPS leads to the upregulation of glutaminase 1 and subsequent elevated ammonia levels and the progression of MASLD to MASH [1,30]. The presence of ectopic reservoirs of LPS-releasing bacteria would be a major health concern.

It should also be noted that the microbiota are sex-dependent, with sex hormones as major drivers of the differences. Women aged 20–45 years old were reported to have a greater microbiota diversity than men of the same age [31]. Whether or not sexual dimorphism in the microbiota is related to the sexual dimorphism in MASLD prevalence [2], referred to above, is unclear.

### 2.2. How Microbiomes Are Characterized

#### 2.2.1. The Blood Microbiome

Today, bacteria are identified using the culture-free methodology of 16S rRNA gene (also referred to as 16S rDNA) sequencing, the history of which we have previously described [11]. The healthy female adult (n = 8) urinary microbiome was reported to comprise the predominant genera of *Lactobacillus*, *Prevotella* and *Gardnerella*, with considerable intersubject variation. The number of operational taxonomic units (OTUs) for individual samples varied substantially, and was in the range of 20–500 [32]. DNA sequences with 97% similarity are clustered together to form an OTU, and a single sequence is taken as representative of that OTU, although this methodology has been subject to criticism [33]. The term “atopobiosis” has been coined to describe microorganisms that appear in places other than their normal locations. The premise of this terminology is that the microbes originate from the gut or the mouth due to dysbiosis [34], a breakdown in the balance between putative species of “protective” versus “harmful” intestinal bacteria and an abnormally permeable mucosal barrier leading to excessive bacterial translocation [35]. Several recent, sequence-based and ultramicroscopic studies have uncovered an authentic blood microbiome [34]. A large study in France measured 16S rDNA concentrations in the blood of 3280 persons without diabetes or obesity at baseline. The 16S rDNA concentration was higher in those who subsequently developed diabetes. No difference was observed regarding obesity. However, the 16S rDNA concentration was higher in those who had abdominal adiposity at the end of follow-up. At the phylum level, Proteobacteria represented 80% to 90% of all bacterial phyla in the blood, both in cases and controls. Within the Proteobacteria phylum, at the genera level, the *Ralstonia* genus was the most prevalent [36].

The tissue microbiota hypothesis was proposed by a French group in 2013, for which it was stated that “the discovery of bacterial DNA within host tissues, such as the liver, the adipose tissue and the blood, which establishes a tissue microbiota, introduces new opportunities to identify targets and predictive biomarkers based on the host to microbiota interaction” [37]. This same group investigated 30 blood donors using 16S rDNA sequencing together with 16S rDNA quantitative PCR (qPCR). Most bacterial DNA was located in the buffy coat (93.74%), with less in erythrocytes (6.23%) and plasma (0.03%). At the phylum level, blood fractions contained bacterial DNA generally from the Proteobacteria phylum (more than 80%), but also from Actinobacteria, Firmicutes and Bacteroidetes. At deeper taxonomic levels, there were conspicuous differences between the bacterial profiles of the different blood fractions [38]. The analysis of 22 common proteins from the blood microbiota suggested that the blood microbiome may have a conserved phylogeny [39], meaning that some aspects of the evolutionary history of the blood microbiota have been maintained throughout their evolution.

A group of 50 patients with severe acute pancreatitis, categorized as uninfected, infected, and septic, was studied using the 16S rDNA sequencing of blood and neutrophil-associated microbiomes. Compared to healthy controls, the blood and neutrophil-associated microbiomes in the patients were significantly altered, with an expansion in Bacteroidetes and Firmicutes as well as a decrease in Actinobacteria. This description of the blood and neutrophil-associated bacterial profiles in severe acute pancreatitis patients offered novel evidence for the existence of the blood microbiome [40]. Others have argued that neutrophils act as a “trojan horse”, protecting intracellular *Staphylococcus aureus* from antibiotics and permitting this organism to travel and infect distant sites [41]. In this context, *Staphylococcus aureus* is not a dormant VBNC organism, but rather an active and virulent one. But this does make the case for a bacterium living intracellularly in blood. Other reports stated that many intracellular pathogenic bacteria survive in vacuolar niches composed of host-derived membranes modified extensively by pathogen proteins and lipids [42,43]. Typical bacteria include *Salmonella typhimurium*, *Listeria monocytogenes* and *Chlamydia trachomatis* [43]. Pathogenic bacteria can create vacuoles in many cell types, but phagocytic cells, such as macrophages, are the most common [42]. These vacuolar niches permit the bacteria to escape humoral immune detection [42,43]. The blood microbiome has been investigated with respect to chronic inflammatory diseases [34], Alzheimer’s dementia [44], chronic kidney disease [45,46], myocardial infarction [47], gastric cancer [48], non-small cell lung cancer [49], hepatocellular carcinoma [50], decompensated liver cirrhosis [51], type 2 diabetes [52], preterm birth [53], rheumatoid arthritis [54], asthma [55] and COVID-19 [56].

#### 2.2.2. The Liver Microbiome

Regarding the liver, the earliest report concerned opisthorchiasis, a parasitic disease caused by *Opisthorchis viverrini*, the Southeast Asian liver fluke that is a major health problem in Thailand, Laos, Vietnam, and Cambodia. This food-borne trematode parasite infects the bile duct, with approximately six million persons infected in northeastern Thailand alone, of which 10% are likely to develop cholangiocarcinoma. The examination of the bile of hamsters with chronic opisthorchiasis revealed a high concentration of Firmicutes, similar to levels in the flukes themselves. Bacteroidetes were low in both bile and the flukes compared to the colon of both normal and infected hamsters. It appeared that the biliary microbiota are derived from the liver flukes, especially as they contained organisms only found in the external environment, such as Cyanobacteria and *Deinococcus* [57]. A subsequent investigation of the liver microbiome of infected hamsters revealed 80 bacterial OTUs, including six phyla and 42 genera. In the chronic *O*. *viverrini*-infected group, bacterial community composition and diversity were significantly increased compared to controls. Sequences of *Fusobacterium* spp. were the most common (13.81%), followed by *Streptococcus luteciae* (10.76%), *Escherichia coli* (10.18%) and *Bifidobacterium* spp. (0.58%). In addition, *Helicobacter pylori* (0.17%) was also identified in the liver of chronic *O*. *viverrini* infections, but not in normal liver [58]. In humans, a 16S rRNA gene sequencing study of 15 Chinese patients with gallstone disease divulged 13 novel biliary bacteria, including *Pyramidobacter piscolens* and *Cellulosimicrobium cellulans* [59]. An investigation in Tomsk, Russia examined the microbiota of bile in patients from western Siberia, who were concurrently infected with a food-borne parasitic worm, the liver fluke *Opisthorchis felineus*. A total of 246 genera were identified in the bile. Those genera found only in *O. felineus*-infected patient bile included *Ruminococcus*, *Oscillospira*, *Anaerostipes*, *Dorea*, *Parabacteroides*, *Aggregatibacter* and *Mycoplana* [60].

The first investigation of the liver microbiome was conducted in *Peromyscus leucopus*, the white-footed mouse, the main reservoir host species of Lyme disease in eastern North America. The *Lactobacillus* genus was found to dominate the liver microbiome. Also detected was a large proportion of individual mice infected with *Bartonella vinsonii arupensis*, a human pathogenic bacteria responsible for endocarditis, as well as *Borrelia burgdorferi*, the pathogen responsible for Lyme disease in North America [61]. In humans, the liver microbiome was said to become oncogenic after exposure to hepatitis B virus (HBV) or alcohol. In patients exposed to HBV that were also alcohol drinkers and had hepatocellular carcinoma (HCC), distinct patterns of both up- and downregulation of certain bacteria were reported. The authors suggest that *Pantoea agglomerans* and *E. coli* 55989 may be important in progression to HCC [62].

As we have previously discussed extensively [1], obesity is a major driver of MASLD. A study was conducted on the liver microbiome from liver biopsies of both healthy lean and healthy obese individuals [63]. Significantly higher amounts of bacterial rDNA (148 vs. 77 16S copies/ng DNA) and lower alpha diversity (the number of types of species measured in a single sample) were found in liver samples from obese relative to lean individuals. A Proteobacteria-augmented metataxonomic signature (metataxonomics refers to the sequencing of the 16S rRNA gene) was found in liver samples from obese individuals. There was a strong correlation between the amount of bacterial rDNA in liver samples and the fatty liver index (FLI) [63], a non-invasive algorithm useful for diagnosing MASLD [64]. Interestingly, differences in the liver microbiome between lean and obese individuals were not reflected in the blood microbiome. The authors concluded that changes in the liver microbiome could constitute an additional risk factor for the development of MASLD [63]. Another group confirmed that the liver microbiome of both mice and humans was distinct from the gut microbiome and enriched in Proteobacteria. Furthermore, the liver microbiota are populated from the gut in a highly selective manner. Additionally, hepatic immunity was found to be dependent on the microbiota, especially the *Bacteroidetes* phylum [65]. For a detailed description of the taxonomy of the human gastrointestinal microbiota, together with the turning points in its history, the reader is directed to Rajilić-Stojanović and de Vos [66].

The divisions of bacteria that dominate human feces and the mucus overlying the intestinal epithelium are Cytophaga–Flavobacterium–Bacteroides (CFB) (for example, the genus *Bacteroides*) and Firmicutes (for example, the genera *Clostridium* and *Eubacterium*), each representing ~30%. Proteobacteria are common but usually not dominant [67]. However, Proteobacteria appear to selectively translocate from the gut lumen into the blood, where they represent the predominant bacterial DNA detected in blood [68]. How Proteobacteria become dominant in the hepatic microbiome is currently unclear. Interestingly, in mild to moderate HCV-related liver fibrosis (Child-Pugh B [69]), Proteobacteria and Alphaproteobacteria were the most abundant in the blood microbiome [70], perhaps suggesting leakage from the damaged liver. Recently, it was reported that the pathogenesis and progression of MASLD were related to immune system dysregulation [71]. Moreover, intrahepatic bacteria, in particular Bacteroidetes, were shown to govern liver immunity through the programming of NKT cells [65]. In another study of 47 overweight or moderately obese MASLD patients and 50 morbidly obese with MASLD, hepatic Bacteroidetes and Firmicutes were over-represented in the morbidly obese patients and Proteobacteria were over-represented in the non-morbidly obese patients. The Proteobacteria were specifically Gammaproteobacteria, Alphaproteobacteria and Deinococcota. These liver bacterial DNA patterns were associated with histological severity [72].

### 2.3. Disrupting the Epithelial Barrier and Translocation to the Liver

There is no consensus as to what constitutes healthy human gut microbiota [73,74], not to mention a healthy blood or liver microbiome. The concept of pathobionts, referring to symbionts that can cause or promote disease only when specific genetic or environmental conditions are altered in the host, has been used for many years [74]. Circumstances under which pathobionts exhibit virulence include impaired host immune defenses and altered microbiota composition. Several microorganisms classified as pathobionts can also exert beneficial functions on their host in different scenarios [74]. *Lactobacillus reuteri* and *Enterococcus gallinarum* are pathobionts that reside in the liver and are believed to have undergone in-host evolution to strains that facilitated their translocation from the gut microbiota [75]. *Lactobacillus* and *Parasutterella* spp. produce indole-3-acetaldehyde and indole-3-acetic acid by tryptophan catabolism, and these molecules act as arylhydrocarbon receptor (AhR) ligands [76] that enhance IL-22 production and repair any damage to the intestinal barrier [77]. In these regards, *Lactobacilli* symbionts function as beneficial microbes in the gut. But when translocated to the liver, *Lactobacillus reuteri* has also been reported to trigger autoimmune hepatitis via the production of the AhR ligand indole-3-acetaldehyde [76]. *Enterococcus gallinarum* has also been reported to trigger autoimmune responses in the liver of both mice and humans, which could be reversed by the antibiotics vancomycin, metronidazole, ampicillin or neomycin [78]. Primary sclerosing cholangitis (PSC) is a chronic inflammatory disease of the liver that often occurs together with ulcerative colitis, highlighting the role of the intestinal epithelial barrier in this disease. *Klebsiella pneumoniae* was identified as the organism in the gut microbiota that disrupted the epithelial barrier and permitted the translocation of several bacterial species. *K. pneumoniae*, *Proteus mirabilis* and *Enterococcus gallinarum* could all be cultured from mesenteric lymph nodes in a mouse model with bacterial-derived hepatobiliary injury. Furthermore, the combination of these three bacteria was observed in patients with PSC [79].

### 2.4. The Part Played by Host Genetics in Shaping the Microbiome

The question of the effect of host genetics on the composition of the liver microbiome has been addressed [80]. These authors focused on variants of host genes that influenced either risk or protection against MASLD histological severity, including *PNPLA3*-rs738409, *TM6SF2*-rs58542926, *MBOAT7*-rs641738 and *HSD17B13*-rs72613567, together with the variant *FGF21*-rs838133, which influenced micronutrient intake. They reported 18 Gammaproteobacteria taxa that associated with the variant alleles, including *Enterobacter*, *Pseudoalteromonas*, *Lawsonella* and *Prevotella 9* and *Staphylococcus*. The abundance of *Tyzzerella* had the strongest association with the polygenic risk score (more than four risk alleles). Details of the tissue microbiota (atopobiotic bacteria) are given in Table 1.

### 2.5. The Part Played by Environmental Factors in Shaping the Microbiome

#### 2.5.1. Water Snails, Fish, Liver Flukes, Ancient and Hardy Bacteria and Biliary Disease

As Table 1 shows, the hepatobiliary system can be populated by a wide range of bacterial DNA, detected by 16S rRNA gene sequencing and 16S rDNA qPCR. Whether or not these findings represent actual live bacteria, for example, VBNC microbes [21], is unclear at present. In bile, bacterial DNA appears only to have been detected under pathological conditions, such as liver fluke infestation both in hamsters [57], and in Russian patients [60] and cholelithiasis patients in China [59]. In the case of hamster bile experimentally infected by the liver fluke *Opisthorchis viverrini*, rDNA from two microbes was reported, from the phylum Cyanobacteria and the genus *Deinococcus*. These results point to the liver fluke as the likely source of the bacteria. *Opisthorchis viverrini* inhabits the bile ducts and gall bladder, where it completes the sexual reproductive stage of its life cycle and is implicated in the development of cholangiocarcinoma and other biliary diseases in infected humans [81]. Interestingly, Cyanobacteria are photosynthetic bacteria, and are commonly referred to as “blue-green algae”, although they are not actual algae [82]. They are aquatic, and it is highly likely that the liver flukes, which conduct the first and second stages of their life cycle in water snails and cyprinid fish (typically carp and minnows), respectively, were the source of the Cyanobacteria DNA detected in hamster bile [57]. Recent investigations on three species of freshwater snails, *Sinotaia quadrata* (endemic to northeast Thailand, see above), *Boreoelona ussuriensis* (endemic to Russia, see above) and *Radix plicatula* (endemic to China and Russia) [83], found that Cyanobacteria comprised 30–40% of the gut microbiome composition of these snails [84], presumably due to their feeding on algae. In addition to Cyanobacteria in the bile of hamsters infected with *Opisthorchis viverrini* liver flukes, *Deinococcus* bacteria were also reported [57]. As has been said, these two organisms were presumably acquired by the liver flukes during stage 1 (water snails) and stage 2 (fish) of their life cycle. Interestingly, *Opisthorchis* has acquired the most ancient known bacterium (Cyanobacterium) and the hardiest bacterium (*Deinococcus*).

Earth was formed around 4.5 billion years ago, and at that time, it had a reducing atmosphere consisting mainly of carbon dioxide, methane and water vapor, as opposed to the present-day atmosphere that consists primarily of nitrogen and oxygen. It has been estimated that life first appeared on Earth 3.8 billion years ago [85,86,87], and for at least 1 billion years, life forms were anaerobic and utilized dissolved minerals for the generation of energy. When Cyanobacteria evolved around 2.7 billion years ago, these photosynthetic microbes were able to split water molecules using the Sun’s energy into oxygen and hydrogen gases, the latter of which would have evaporated into space. The emergence of an oxygen atmosphere, known as The Great Oxidation Event and The Oxygen Holocaust, is thought to have resulted in the advent of a dominant Cyanobacteria, with the mass extinction of anaerobic life forms, around 2.1 to 2.4 billion years ago [88].

Of approximately 50 species of *Deinococcus* known, *Deinococcus radiodurans* is able to endure an acute dose of 5000 Gy (500,000 rad) of ionizing Gamma radiation and up to 15,000 Gy with 37% viability [89]. In addition to tolerating ionizing radiation, *Deinococcus radiodurans* can withstand extreme temperatures, vacuum, oxidation and desiccation [90]. This is due to its complex cell envelope encapsulated within a hyperstable surface layer [91]. It is described in the Guiness Book of World Records as “the most radiation-resistant lifeform” [92]. It is bizarre that the *Opisthorchis viverrini* liver fluke carries both the most ancient and the least destructible bacteria as passengers, which it can transfer into mammalian bile. *Opisthorchis viverrini* has been categorized as a group 1 carcinogen since 1994 by The International Agency for Research on Cancer (IARC), the only eukaryote known to cause cancer, specifically cholangiocarcinoma [93]. What role, if any, is played by these passenger bacteria in the pathobiology of cholangiocarcinoma and other biliary diseases appears not to have been investigated.

#### 2.5.2. Diet and the Microbiome

To investigate the effects of diet on the human gut microbiota, humanized gnotobiotic mice were created by the gavage of a freshly produced healthy human stool sample. Mice were maintained on a low-fat, plant polysaccharide-rich (LF/PP) diet, and, after one month, half the mice were switched to a high-fat, high-sugar western diet. The characterization of the taxa present in the gut microbial communities of the two groups of humanized gnotobiotic mice was conducted using 16S rRNA gene qPCR. The western diet-associated humanized mouse microbiota showed an increased representation of the Erysipelotrichi class of bacteria within the Firmicutes phylum relative to mice fed the LF/PP diet. Phylogenetic analysis has indicated that organisms with increased representation were most closely related to *Clostridium innocuum*, *Eubacterium dolichum* and *Catenibacterium mitsuokai*, all Erysipelotrichi previously isolated from the human gut. Surprisingly, a clear shift in rRNA gene content was evident one day after the switch to the western diet [94]. Clearly, the type of diet we consume has a major effect on the gut, and therefore tissue microbiomes, and plays a significant role in the development of obesity, type 2 diabetes mellitus (T2DM) and MASLD, as will be seen below. In mice, a diet high in saturated fat such as palm oil increased steatosis, decreased microbial diversity, and increased the Firmicutes to Bacteroidetes ratio, potentially increasing energy harvest from the gut [95]. Another study found that mice fed a diet of animal fat, high in saturated fats and cholesterol, had increased hepatic TLR4 activation and white adipose tissue inflammation, with reduced insulin sensitivity compared to mice fed fish oil. Animal fat-fed animals had significantly decreased phylogenetic diversity and increased levels of *Bacteroides*, *Turicibacter* and *Bilophila*, whereas fish-oil-fed mice had increased levels of Actinobacteria (*Bifidobacterium* and *Adlercreutzia*), lactic acid bacteria (*Lactobacillus* and *Streptococcus*), Verrucomicrobia (*Akkermansia muciniphila*), Alphaproteobacteria and Deltaproteobacteria [96]. The modification of the human diet, for example via the addition of fresh fruit such as grapes, can influence the composition of the gut microbiota [97].

## 3. The Microbiota and MASLD

The role of the intestinal microbiota in MASLD has been reviewed [98]. The gut microbiota contribute to multiple facets of what has been described as “metabolic inflammation”, a hallmark of metabolic diseases such as obesity, T2DM and MASLD [99]. Intestinal bacteria, as has been discussed, can breach the intestinal epithelial barrier to reach the liver and adipose tissue, and provoke metabolic inflammation [99]. Healthy subjects show a diverse composition of the intestinal microbiota and an intact intestinal epithelial barrier, which prevents the infiltration and systemic propagation of bacteria and their mediators [99,100]. Our current understanding is undoubtedly due to the advent of genetic tools referred to above, and the metagenomic revolution of the last 20-plus years, which permit the characterization of the composition and function of microbiomes from different parts of the body, permitting linkage to diseases [100]. The gut microbiota is a clear contributor to both the development of MASLD and its progression to MASH. It was suggested 15 years ago that the modification of the gut microbiota “may represent a new (though as yet unproven) strategy in the management of patients” with MASLD or MASH [5]. First, it is necessary to consider the microbiota in further detail.

### 3.1. The Role of Bacteriophages in the Microbiota

In the gut microbiota, bacteria are not the sole players; archaea, viruses, fungi and bacteriophages (phages) may also influence microbiota–host interactions, especially as phages outnumber bacteria in the gut by a ratio of ten-to-one. The majority of the gut virome comprises bacteriophages, and this has been referred to as the phageome [100,101]. A detailed account of intestinal phages and their relationship with bacteria has been given [102]. Fresh fecal samples from a number of healthy individuals were analyzed both by 16S rRNA amplicon sequencing for the bacteriome and virus-like particle (VLP) DNA extraction, followed by the shotgun sequencing of VLP nucleic acid for determining phageome sequences. Interestingly, individual specificity was greater for the phageome than the bacteriome [101]. Recently, great strides have been made to characterize components of the phageome [103,104]. For example, temperate (lysogenic) prophages (phages that integrate their genomes into bacterial or archaeal chromosomes) have been newly described [104]. Previous studies have indicated that temperate phages are beneficial to their susceptible bacterial hosts by introducing additional genes to bacterial chromosomes, including antibiotic resistance genes, creating a mutually beneficial relationship [105]. A group in China analyzed 43,942 human gut-derived bacterial genomes (439 species of 12 phyla) and identified 105,613 prophage regions in ~92% of them [104]. These workers also observed that ~72% of prophages had previously unreported genomes, and they illuminated ~6.5 to 9.5% of the individual intestinal “viral dark matter” [104].

A lytic bacteriophage cocktail comprising 15 distinct temperate phages that together targeted *Listeria monocytogenes*, *Salmonella* spp., and Shiga toxin-producing *Escherichia coli* has been investigated [106]. *L. monocytogenes* is a species of pathogenic bacteria that causes the infection listeriosis. It is a facultative anaerobe and is therefore capable of surviving in the presence or absence of oxygen. It can grow and reproduce inside the host’s cells and is one of the most virulent foodborne pathogens. Twenty to thirty percent of foodborne listeriosis infections in high-risk individuals may be fatal [107]. It was reported that the phage cocktail was as effective as the antibiotic ampicillin in reducing *L. monocytogenes* in both a model ileum and colon environment. Moreover, the phage cocktail did not inhibit commensal bacteria, whereas ampicillin treatment led to dysbiosis [106]. Whether or not these phages can inhibit pathogenic bacteria inside human cells in vivo remains to be proven.

Bacteriophages, in particular so-called tailed phages, have dsDNA genomes of 40–50 kb and around 50 genes, which occupy about 90% of the genome sequence [108]. Tailed phages are remarkably abundant. As an example, there are typically ~10^7^ tailed phage particles per milliliter in coastal sea water [109]. Furthermore, the global population is estimated to be in excess of 10^30^, more than every other organism on Earth, including bacteria [108]. Phages differ considerably in the bacterial hosts that they infect. Their host range is governed by the specific structures that they use to target bacterial cells. Tailed phages use a broad range of receptor-binding proteins, such as tail fibers, tail spikes and the central tail spike, to target their cognate bacterial cell surface receptors. For example, the Enterobacteriaceae that comprise 30 genera and over 100 species of Gram-negative bacteria, including *E. coli*, *Klebsiella* and *Salmonella*, are infected by *Myoviridae* T4 and Mu phages [110]. These phages have receptor binding proteins at the tips of their tail fibers that recognize host receptors, such as LPS, leading to the eventual puncturing of the bacterial cell wall and injection of the phage nucleic acid [111].

Surprisingly, bacteriophage therapy has been in practice for over 100 years. The Franco-Canadian microbiologist Félix d’Hérelle is credited with the co-discovery and naming of bacteriophage, a bacterial virus, at the Pasteur Institute in Paris [112]. In 1917, d’Hérelle and other microbiologists isolated phages able to kill then-known pathogenic bacteria, such as *Shigella dysenteriae*, *Salmonella typhi*, *Escherichia coli*, *Pasteurella multocida*, *Vibrio cholerae*, *Yersinia pestis*, *Streptococcus species*, *Pseudomonas aeruginosa* and *Neisseria meningitis* [113]. In the treatment and prevention of large epidemics, phage suspensions were administered by both topical application [114] and systemic administration through oral routes and/or injection [115,116,117,118,119]. These applications were successfully used to treat Staphylococcal infections of the skin, bone, eye, and others [120,121,122]; intestinal pathologies such as typhoid, dysentery and cholera [123,124,125]; and systemic infections such as sepsis [126,127]. In addition to the work of these various investigators from 1924 to 1949, d’Hérelle himself continued to work on bacteriophages. One particular investigation of note involved a collaborator, Major RH Malone of the Indian Medical Service at the Central Research Institute (formerly the Pasteur Institute of India) in Kasauli, Himachal Pradesh, India. d’Hérelle and Malone isolated bacteriophages virulent for *Vibrio cholerae* from the stool of patients freshly admitted to the Campbell Hospital, Calcutta (now Kolkata). They then correlated the levels of the bacteriophage isolated with clinical outcomes. Six patients with no detectable or a “feeble” fecal bacteriophage count died from cholera within 24 h, while two cases with a “strong bacteriophage at the moment of admission” made prompt recoveries. In between these two extremes, 15 cases with a weak or fluctuating fecal bacteriophage level all made a delayed recovery [128]. It must be stated that no experimental methodology or reference to it was included in this report. All this notwithstanding, the Pasteur Institute of India was established in 1900 by its first director Major David Semple (later Lieutenant-Colonel Sir David Semple; 1856–1937) for the treatment of dog bites and related cases. Semple developed the first anti-rabies vaccine, and the Institute was clearly competent in virology and immunology at the time of the investigations of d’Hérelle and Malone.

By the end of World War II, western interest in phage therapy had cooled considerably. Phage therapy had been employed by the militaries of the US’s enemies, Germany and Japan, and continued to be developed in the Soviet Union, and, according to the medical historian William C. Summers, in the immediate post-McCarthy days of the cold war, was seen as un-American [129]. Throughout Georgia, Russia, Ukraine, Belarus, and Azerbaijan, due to the relationship between d’Hérelle and his Georgian colleagues, work on bacteriophages flourished [130]. The results of clinical trials in dermatology, ophthalmology, urology, stomatology, pediatrics, otolaryngology, and surgery in the 1930s and 1940s were published in Russian, and therefore not readily available to western investigators. The unequivocal demonstration of the action of phages on bacteria awaited the development by Siemens of a commercial electron microscope (EM) in Germany in 1939. For his work on the development of the first electron microscope, Ernst Ruska shared the 1986 Nobel Prize in Physics [131]. The first EM images of bacteriophage lysis of bacteria were published by Ruska in German in 1940 [132], and so did not become available in the west until his findings were republished in France in 1942 [133].

Despite these developments, demonstration of the antibacterial efficacy of the first sulfonamide Prontosil in 1932 (for which Gerhard Domagk was awarded the 1939 Nobel Prize in Physiology or Medicine [134]) and discovery of the first antibiotic penicillin (for which Alexander Fleming, Howard Flory and Ernst Chain shared the 1945 Nobel Prize for Physiology or Medicine [135]) further quashed any residual interest in d’Hérelle’s phage therapy. As stated by Summers [129], “phage therapy, even if it had been unambiguously effective, was just too complicated for the state of American medicine in the 1940s. The therapeutic niche for phage became occupied by the more ‘fit’ antibiotics”. Fast forward to the present past, the emergence of antibiotic resistance has rekindled western interest in d’Hérelle’s original work [130,136,137]. The WHO stated the following in June, 2024: “Bacteriophages, also known as phages, are viruses that selectively target and kill bacteria. These natural biological entities are ubiquitous in the environment and can destroy bacteria that are resistant to medicines such as antibiotics. Phages offer a promising alternative or adjunct to antibiotics” [138].

### 3.2. Bacteriophages and MASLD

The usage of bacteriophages to kill drug-resistant bacteria associated with liver disease has been proposed [139]. So-called “bacteriophage therapeutics” [140], in the context of antibiotic resistance, was first used clinically to successfully treat a disseminated resistant *Acinetobacter baumannii* infection [140] and an aortic graft infected with *Pseudomonas aeruginosa* [141]. Many investigators have since evaluated bacteriophage therapy for liver diseases. One example involved humanized mice that had been colonized with bacteria from the feces of patients with alcoholic hepatitis. Hepatocyte death and liver injury were due to an exotoxin secreted by *Enterococcus faecalis*. These investigators isolated four distinct podophages of the virulent *Picovirinae* group from sewage water, and these were able to lyse *E. faecalis*. It was reported that gavage of the humanized gnotobiotic mice with a cocktail of these four bacteriophages reduced the levels of exotoxin in the liver and attenuated hepatitis and liver injury [142]. In patients, intestinal viral taxa were altered in fecal samples from patients with alcoholic hepatitis and were associated with disease severity and mortality [143]. Similarly, in the study of fecal viromes from patients with MASLD and control individuals, histological markers of MASLD severity were associated with significant decreases in viral diversity and the proportion of bacteriophages [144]. The relationship between numbers of phages and their host bacteria was investigated. *Lactococcus* phages were the most abundant phages, but the level of *Lactococcus* was comparably low. Bacteroides was highly abundant, whereas the corresponding Bacteroides phages had low abundance and were detected in only a few patients. These findings are an echo of the work in India conducted by d’Hérelle and Malone on cholera almost a century earlier, whereby there appeared to be a positive correlation between levels of fecal bacteriophage and patient survival [128]. In the MASLD report, viral diversity did not correlate with bacterial diversity. The authors concluded that the intestinal virome could contain information to identify patients with MASLD at risk of future liver-related complications [144]. It is said that phages and bacteria within the gut microbiome persist in long-term stable coexistence [145].

Experiments have been conducted to investigate one of the most abundant phages in the gut, the crAss-like phages (*Crassvirales*), which infect members of the Bacteroidales, in particular *Bacteroides* [145]. The discovery in silico of crAssphage, a highly human-specific resident of the gut, has been of utmost importance, as it is the only comprehensive marker of human fecal pollution so far described [146,147] that is also stable in the environment [148]. Using a multiomic strategy involving transcriptomics, proteomics and metabolomics, multiple resistance mechanisms were reported. The authors concluded that knowledge of the complexities of phage–bacteria interactions was essential for designing effective phage therapies and improving human health through targeted microbiome interventions [145]. The Gut Phage Database has been developed and contains ~142,000 non-redundant viral genomes assembled from 28,060 human gut metagenomes spanning 23 countries from all continents. Interestingly, a highly prevalent phage clade with features indicative of a crAssphage was found [149].

In the US, Hispanics are at the highest risk of MASLD, which is often concurrent with obesity and diabetes [150]. This disparity is almost entirely attributable to Mexican Americans [151], with moderate and severe hepatic steatosis occurring twice as frequently in Mexican Americans as European Americans [152]. The gut phageome in Mexican Americans in South Texas was investigated by the stool shotgun metagenomic sequencing of 340 subjects, who had concurrently been screened for liver steatosis by transient elastography. The enrichment of *Inoviridae* was associated with both diabetes and liver steatosis. *Inoviridae* comprise 21 genera and 27 species, for example, *Escherichia virus M13* [153]. Diabetes was further associated with the enrichment of predominantly temperate *Escherichia* phages, some of which possessed virulence genes. The enrichment of the globally prevalent *Crassvirales* phages was observed in liver steatosis [151]. Interestingly, work conducted with respect to the poultry meat industry examined chicken livers for bacteria and phages because of the known occasional contamination with pathogenic bacteria such as *Campylobacter* and *Salmonella*. Bacteria and phages that were able to infect *E. coli* were found in all samples studied. The phages carried several antibiotic resistance genes and, moreover, there was a correlation between chicken liver phages and fecal phages, suggesting that bacteriophages translocate from the gut to the liver, as do bacteria. The authors proposed that the liver may therefore constitute a reservoir of antibiotic resistance genes [154].

There are relatively few, if any, clinical studies investigating whether or not phage therapy can be used to treat MASLD. One has been conducted in an animal model, in which mice colonized with high-ethanol-producing *Klebsiella pneumoniae* developed steatohepatitis. Phage therapy against ethanol-producing *K. pneumoniae* alleviated steatohepatitis [155] and was proposed as a potential treatment for MASLD associated with high-ethanol-producing *Klebsiella pneumoniae* [156,157,158]. On the consumption of alcohol by MASLD patients, it was reported that the intestinal phageome of MASLD patients who consume low to moderate amounts of alcohol is significantly different from that of those who do not, and many features of the intestinal phageome of alcohol-consuming MASLD patients resemble those of the phageome of alcohol use-related disease patients [159]. Recent advances emphasize the role of the gut phageome in the development of metabolic disorders such as obesity, T2DM, MASLD, and cardiovascular diseases [160]. Today, due to the growing threat of antibiotic-resistant bacterial pathogens, phage therapy is experiencing a resurgence. As has been stated, “Currently, phage therapy for metabolic diseases is expected to develop into a pre-defined phage-based medicinal product. However, numerous unknowns remain, and much research is needed before its clinical application” [160].

### 3.3. Microbiota Metabolism and Its Impact on MASLD

#### 3.3.1. Gut Microbiota Metabolism of Bile Acids

**Figure 1:**
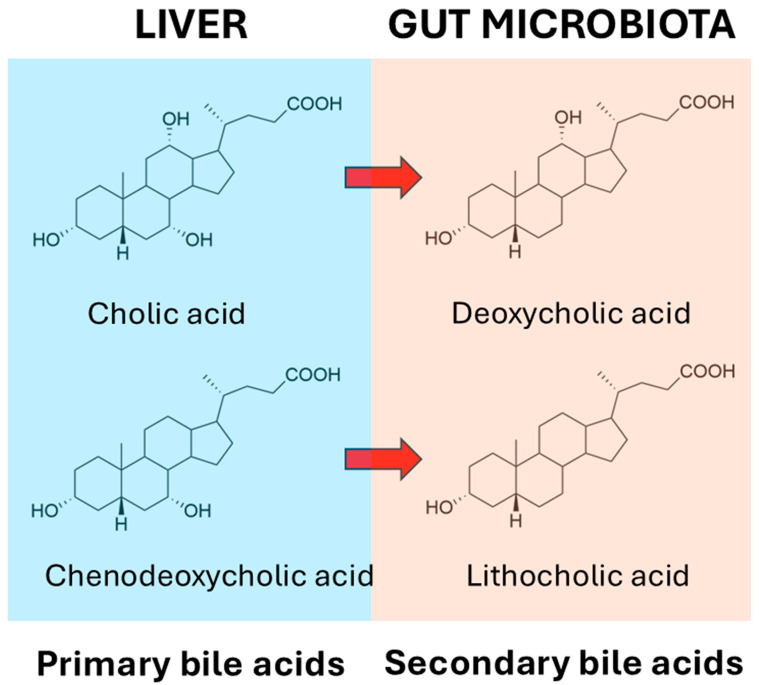
Deconjugation of glycocholic acid and taurocholic acid by gut microbiota.

It has long been known that the gut microbiota deconjugate primary bile salts and convert them into primary bile acids. The deconjugation reactions for glycocholate and taurocholate are shown in Figure 1, and these reactions apply also for glycochenodeoxycholate and taurochenodeoxycholate.

**Figure 2:**
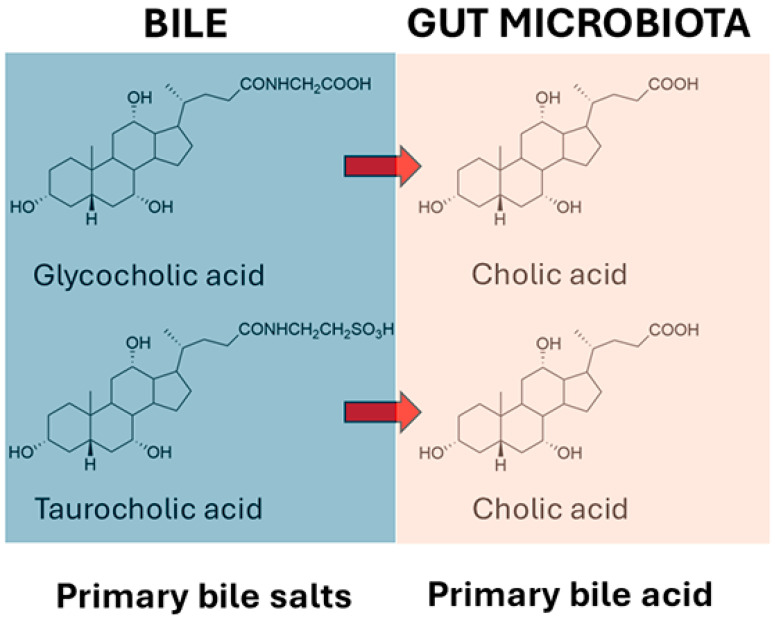
Dehydroxylation of primary bile acids to secondary bile acids by the microbiota.

At the outset, it should be stated that the relationship between bile acids and the microbiome is complex, and that this complexity adds numerous further levels of importance to our understanding of gut bacterial bile acid metabolism. For example, while intestinal bacterial bile acid metabolism impacts the bile acid profile, there is also a bidirectional effect of bile acids on the gut microbial profile, as some bacteria are more able to survive in the presence of bile acids than others [161]. But what are these bacteria? When the liver synthesizes primary bile acids from cholesterol, it first conjugates them with glycine or taurine into bile salts, prior to transport into the bile. These primary bile acid conjugates are first deconjugated by gut microbiota, prior to 7α-dehydroxylation to secondary bile acids, together with a number of other reactions such as dehydrogenation and reconjugation. According to Wise and Cummings [161], many Gram-positive and Gram-negative bacteria are able to deconjugate bile salts to bile acids using bile acid hydrolases, and these bacteria include *Bacteroides*, *Clostridium*, *Lactobacillus*, *Bifidobacterium*, and *Listeria* [162,163]. The recent literature suggests that there are likely bacteria that are capable of 7α-dehydroxylation that are yet to be discovered [164]. The deconjugated bile acids can then undergo a panoply of metabolic reactions, the major representatives of which are sulfation, dehydrogenation, and dehydroxylation [165]. The principal reaction is the removal of the 7α-hydroxy group from cholic acid, forming deoxycholic acid, and from chenodeoxycholic acid, forming lithocholic acid (Figure 2). Over 50 years ago, workers at the Rockefeller University, New York provided persuasive evidence that the incapacitation of the microbiota by the administration of the antibiotics neomycin, kanamycin or chloramphenicol resulted in the highly significant impairment of cholic acid metabolism by fresh homogenized human stool. In addition, a significant decline in serum cholesterol levels was observed, but not in all of the 25 patients investigated. In a minority of patients where serum cholesterol levels were not lowered by antibiotic treatment, the 7α-dehydroxylation of cholic acid failed to occur. The correlation coefficient between undegraded cholic acid and the percent decrease in serum cholesterol concentrations *(r* = 0.732) was statistically significant (*p* < 0.001). The authors posited that serum cholesterol levels might be controlled in part by the prevalence within the gastrointestinal tract of bacteria capable of the 7α-dehydroxylation of primary bile acids [166].

This reported interpatient variability in 7α-dehydroxylation [166] is consistent with our current understanding of microbiota variability from a comparison of 11 abundant gut bacterial species, which showed that the gene contents of strains from the same species differed, on average, by 13% between individuals [167]. Moreover, 53 bacterial species were found to be capable of detecting MASLD with robust diagnostic accuracy (area under the receiver-operator curve (AUC) = 0.97). In addition, eight markers of secondary bile acid synthesis were identified in MASLD, and these included elevated abundance of 7α-hydroxysteroid dehydrogenase, 3α-hydroxysteroid dehydrogenase, and bile acid-coenzyme A ligase. Genes for secondary bile acid synthesis were dominant for *Bacteroides ovatus* and *Eubacterium biforme* in MASLD [168]. A systematic identification of secondary bile acid production genes has been conducted in human, pig, dog, cat, mouse guts, together with human skin, vagina, nose and mouth, and various environmental sources, the most prevalent of which was wastewater. For the bile salt hydrolase (BSH) genes in gut microbiota, the prevalence can be ranked as pig > human > mouse > cat > dog. In contrast, bile acid-inducible dehydroxylation genes in gut microbiota rank as dog > cat > mouse ≈ human > pig. Finally, hydroxysteroid dehydrogenase genes in gut microbiota rank as dog ≈ cat ≈ mouse > human > pig. For non-intestinal human microbiota, all three groups of genes were found in smaller proportions, ranked as skin > vagina > nose > mouth [169].

Mice fed a high-fat western diet developed MASLD, which led to increased secondary bile acids associated with *Clostridium* proliferation [170]. It was recently reported that mice with MASH had 3.5-fold lower 7α-dehydroxy bile acid content than controls, but had a normal gut microbiota 7α-dehydroxylating capacity. They identified an increased level in bile acid 7α-rehydroxylation mediated by liver CYP2A12 and CYP2A22 enzymes (4.0-fold higher), which reduced secondary 7α-dehydroxylated bile acid levels in MASH [171]. In humans, over 117 genera in 12 phyla of the microbiota harbor BSH genes [172], but only a few *Clostridium* species, namely, *C. scindens*, *C. hylemonae*, *C. hiranonis*, and *C. leptum*, are able to conduct 7α-dehydroxylation [173]. Many other *Clostridium* species do not possess this activity. It has been estimated that the few identified bacterial strains that can perform bile acid 7α-dehydroxylation are the Gram-positive anaerobes found in low abundance. These strains were estimated to represent only one one-millionth of the total gut microbes in humans [173]. Clearly, other gut microbes that can carry out bile acid 7α-dehydroxylation await characterization and the determination of their role in MASLD.

A meta-analysis of 19 studies with 154,807 individuals (43,229 MASLD patients and 111,578 healthy controls) has been reported, in which it was found that total bile acids were higher in MASLD patients than in healthy controls. Furthermore, 9 of the 15 measured bile acids were increased in MASLD patients, especially ursodeoxycholic acid, taurocholic acid, chenodeoxycholic acid, taurochenodeoxycholic acid, and glycocholic acid. Taurocholic acid, taurodeoxycholic acid, taurolithocholic acid, and glycolithocholic acid had the potential ability to differentiate MASLD from MASH [174]. Although deoxycholic acid and lithocholic acid are the most abundant secondary bile acids, ~50 different secondary bile acids have been detected in human feces [175].

#### 3.3.2. Gut Microbiota Metabolism of Tryptophan

**Figure 3:**
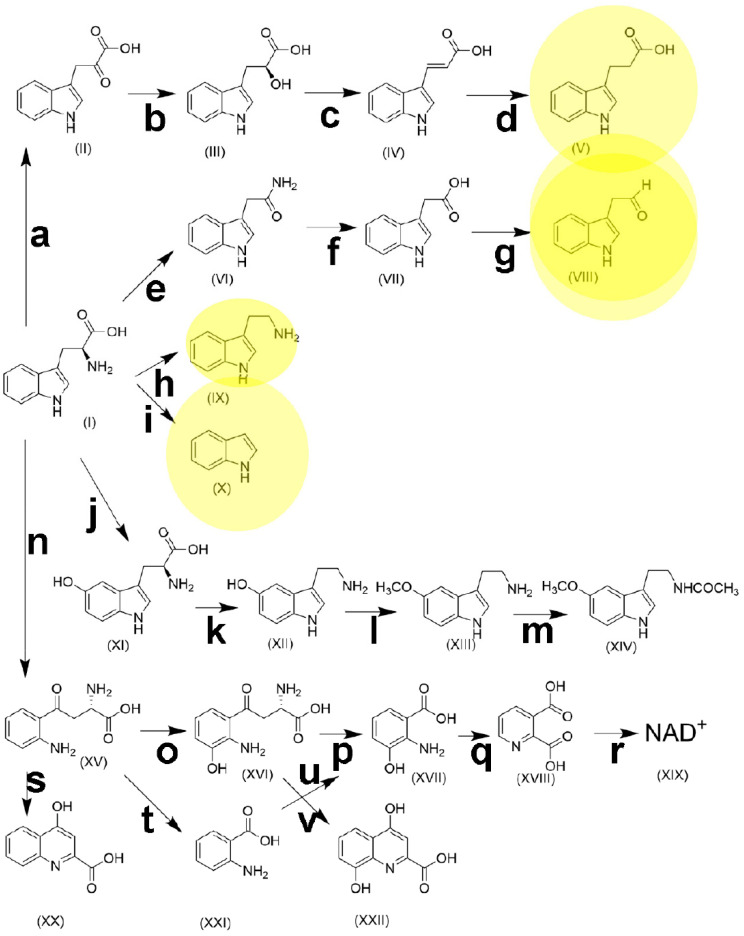
The metabolic pathways of tryptophan. (I), tryptophan; (II), indole-3-pyruvic acid; (III), indole-3-lactic acid; (IV), indole-3-acrylic acid; (V), indole-3-propionic acid; (VI), indole-3-acetamide; (VII), indole-3-acetic acid; (VIII), indole-3-acetaldehyde; (IX), tryptamine; (X), indole; (XI), 5-hydroxytryptophan; (XII) 5-hydroxytryptamine (serotonin); (XIII), 5-methoxytryptamine; (XIV), *N*-acetyl-5-methoxytryptamine (melatonin); (XV), kynurenine; (XVI) 3-hydroxykynurenine; (XVII), 3-hydroxyanthranilic acid; (XVIII), quinolinic acid; (XIX), nicotinamide adenine dinucleotide; (XX), kynurenic acid; (XXI), anthranilic acid; (XXII), xanthurenic acid. **a**, aromatic amino acid transaminase [EC 2.6.1.57]; **b**, indolelactate dehydrogenase [EC 1.1.1.110]; **c**, phenyllactate dehydratase [EC 4.2.1.175]; **d**, 3-(aryl)acrylate reductase [EC 1.3.8.15]; **e**, tryptophan 2-monooxygenase [EC 1.13.12.3]; **f**, indoleacetamide hydrolase [EC 3.5.1.-]; **g**, indole-3-acetaldehyde oxidase [EC 1.2.3.7]; **h**, L-tryptophan decarboxylase [EC 4.1.1.105]; **i**, tryptophanase [EC 4.1.99.1]; **j**, tryptophan hydroxylase [EC 1.14.16.4]; **k**, aromatic L-amino acid decarboxylase [EC 4.1.1.28]; **l**, hydroxyindole *O*-methyltransferase [EC 2.1.1.4]; **m**, arylalkylamine *N*-acetyltransferase [EC 2.3.1.87]; **n**, tryptophan 2,3-dioxygenase [EC 1.13.11.11] + indoleamine 2,3-dioxygenase; [EC 1.13.11.52]; **o**, kynurenine 3-monooxygenase [EC 1.14.13.9]; **p**, 3-hydroxy-L-kynurenine hydrolase [EC 3.7.1.3]; **q**, 3-hydroxyanthranilate 3,4-dioxygenase [EC 1.13.11.6]; **r**, quinolinic acid phosphoribosyltransferase [EC 2.4.2.19] + nicotinate-nucleotide adenylyltransferase [EC 2.7.7.18] + nicotinamide adenine dinucleotide synthetase [EC 6.3.1.5]; **s**, kynurenine aminotransaminase [EC 2.6.1.7]; **t**, kynureninase [EC 3.7.1.3]; **u**, anthranilate 3-monooxygenase [EC 1.14.14.8]; **v**, kynurenine aminotransferase [EC 2.6.1.7]. Yellow shading represents terminal metabolites of gut microbiota pathways.

L-Tryptophan is an essential α-amino acid required for protein synthesis in humans and animals. Dietary tryptophan undergoes an extensive and complex metabolism along several pathways, resulting in many bioactive molecules, such as serotonin, melatonin, kynurenine, and NAD^+^ (Figure 3). It has been estimated that 95% of tryptophan metabolism proceeds to kynurenine [176]. As the sole precursor of serotonin, experimental research has shown that the role played by tryptophan in brain serotonin synthesis is an important factor involved in mood, behavior, and cognition [177]. 

The principal dietary sources of tryptophan include oats, bananas, dried prunes, milk, tuna fish, cheese, bread, chicken, turkey, peanuts, and chocolate. The recommended daily allowance for adults is 250 to 425 mg/day; however, many Americans consume 900 to 1000 mg/day [177]. Most dietary tryptophan is absorbed in the ileum, although a proportion reaches the colon where it is metabolized by the intestinal microbiota [178]. Many of the metabolic pathways are carried out in the liver and recapitulated by the microbiota, as shown in Table 2.

The administration of indole to mice with HFD-induced MASLD resulted in a significant improvement in hepatic steatosis and inflammation. The authors concluded that elevating dietary tryptophan to increase microbiota-generated indole levels may represent an effective approach for preventing and treating MASLD [189]. In the HFD-induced MASLD mouse model, the disease state had a major effect on the composition of fecal microbiota, with significant increases in the abundances of *Bacteroides*, *Muribaculaceae, Allobaculum, Paramuribaculum* and *Ruminococcaceae*, alongside notable decreases in the abundances of *Ligilactobacillus, Lactobacillus* and *Enterococcus faecalis HT002* within the MASLD group. Specifically, *Ileibacterium valens*, *Ruminococcaceae*, *Duncaniella frettoniella*, *Paramuribaculum*, *Coriobacteriaceae*, *Marmorella UCG_002* and *Olsenella* were identified as characteristic organisms associated with MASLD. Furthermore, these authors found key metabolites significantly enriched in tryptophan metabolism, and focused on the principal kynurenine pathway (**n**, **o**, **p**, **q**, **r** in Figure 3) [190], which is modulated by indoleamine 2,3-dioxygenase (IDO) (**n** in Figure 3). This enzyme is upregulated by pro-inflammatory molecules such as INF, IL-6 and LPS. A higher activity of IDO is associated with increased inflammation and fibrosis in MASLD [191]. An investigation of 15 tryptophan metabolites from the kynurenine, indole and serotonin pathways (see Figure 3) in 76 patients with normal liver, simple steatosis or MASH found a decrease in indole-3-acetic acid (VII) and indole-3-propionic acid (V) in relation to obesity. In addition, an elevated serum tryptophan concentration was associated with MASLD. Pathway fluxes demonstrated an induction of tryptophan catabolism via the serotonin pathway in simple steatosis subjects, and into the kynurenine pathway in MASH [192]. Obesity is found in 50% to 90% of MASLD patients, and we have written extensively about obesity as a precursor of MASLD [1]. It has been reported that both kynurenine and microbiota-mediated indole routes of tryptophan metabolism were altered in obese subjects, relative to non-obese controls, as reflected in a higher kynurenine/tryptophan ratio and lower levels of serotonin and indoles [193]. Similar findings were reported for 374 pregnant women, where BMI correlated negatively with plasma tryptophan concentration but positively with plasma kynurenine concentration and the kynurenine/tryptophan ratio. Plasma tryptophan was significantly lower, and the kynurenine/tryptophan ratio was significantly higher in women with obesity [194]. Obesity and MASLD are clearly significantly influenced by tryptophan metabolism by both the host and microbiota. A study of 106 severely obese individuals identified metabolic profiles associated with MASLD progression to MASH. Using nontargeted metabolomics undertaken with UPLC-QTOFMS [1,195], indole-3-propionic acid (V) was negatively correlated to fibrosis, and tryptophan was positively correlated to lobular inflammation and hepatocyte ballooning [196].

It has been suggested that bile acids, short-chain fatty acids, trimethylamine *N*-oxide, and tryptophan metabolites due to microbiota metabolism are closely associated with the onset and progression of MASLD [197]. Moreover, a meta-analysis revealed that increased *Escherichia*, *Prevotella*, and *Streptococcus* genera and decreased *Coprococcus*, *Faecalibacterium*, and *Ruminococcus* genera represent the universal intestinal microbiota signature of MASLD [198]. According to Table 2, *E. coli* is the only tryptophan-metabolizing gut microbiota member with this universal signature of MASLD. *E. coli* is responsible for the metabolism of tryptophan to indole, indole-3-aldehyde, indole-3-acetic acid, and indole-3-propionic acid, and therefore these indoles are likely to play a role in MASLD. It was recently reported that fecal *Bifidobacterium bifidum* was increased in parallel with the progression of MASLD, and that tryptophan increased while indole-3-acetic acid and indole-3-propionic acid decreased. These findings suggest that the metabolism of tryptophan by the microbiota to these two indolic acids was impaired in MASLD. To test this, these authors used a mouse model with western diet (WD)-produced MASLD, and administered tryptophan, indole-3-acetic acid, indole-3-propionic acid or *Bifidobacterium bifidum,* all individually by gavage. The administration of *B. bifidum* alleviated WD-induced hepatic steatosis and inflammation in mice [199]. Indole-3-propionic acid appears to be important in delaying or attenuating the symptoms of MASLD [200]. It may accomplish this by inhibiting gut microbiota dysbiosis and the leakage of endotoxin from the gut [201]. Additionally, indole-3-acetic acid has also been reported to alleviate MASLD in HFD-fed mice [202]. The administration of another tryptophan metabolite, kynurenic acid, to mice fed a high-fat diet ameliorated hepatic lipid accumulation and decreased the expression of lipogenic genes, as well as ER stress [203]. Kynurenic acid was predicted in silico to be produced by several gut microbiota genera, including *Clostridium*, *Burkholderia*, *Streptomyces*, *Pseudomonas*, and *Bacillus* [204].

In summary, the gut microbiota represent a key player in the development of MASLD by mechanisms such as dysbiosis and intestinal epithelial leakage, which permits bacterial endotoxin egress into the portal supply of the liver. In contrast, the microbiota can biotransform dietary tryptophan into metabolites that have a beneficial effect on MASLD by reversing many of these mechanisms.

#### 3.3.3. Microbiota Metabolism of Histidine

**Figure 4:**
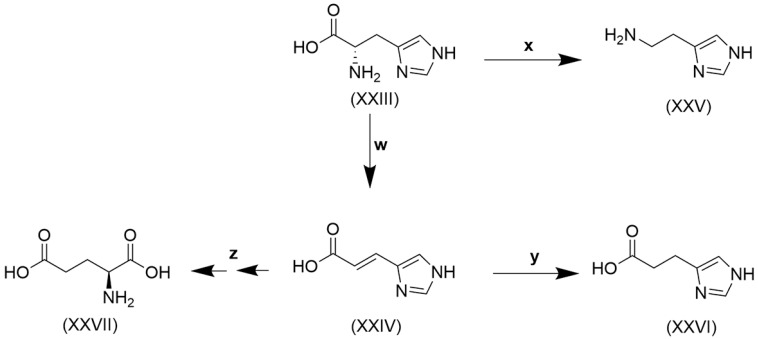
Catabolism of histidine. (XXIII), histidine; (XXIV), urocanate; (XXV), histamine; (XXVI), imidazole propionate; (XXVII), glutamate. **w**, histidine ammonia lyase [EC 4.3.1.3]; **x**, histidine decarboxylase [EC 4.1.1.22]; **y**, urocanate reductase [EC 1.3.99.33]; **z**, urocanate hydratase [EC 4.2.1.49] + imidazolone propionase [EC 3.5.2.7] + formiminoglutamase [EC 3.5.3.8].

Histidine, like tryptophan, is another of the nine essential amino acids that cannot be synthesized by humans to satisfy the need for metabolic reactions [205]. Histidine is obtained from the diet mainly in the form of proteins. Its content in proteins of animal sources, like meat, chicken, and fish, is 25–30 mg/g, and in plant proteins, like soybean, kidney beans, peas, oat, and wheat, it is 20–30 mg/g [206]. Dietary histidine undergoes catabolism by two major pathways, shown in Figure 4.

Histidine (XXIII) conversion to urocanate (XXV) and on to glutamate (XXVII) was first reported in the aquatic and terrestrial bacterium *Pseudomonas fluorescens* [207,208], and subsequently urocanate formation in unfractionated guinea pig liver homogenate was identified as the principal metabolic reaction of histidine [209]. The formation of histamine (XXV) from histidine by reaction **x** (Figure 4) was shown to occur in several members of the gut microbiota, including *Klebsiella aerogenes*, *Fusobacterium varium*, *Clostridium perfringens*, *Limosilactobacillus reuteri* and *Morganella morganii* [210]. Moreover, all the reactions depicted in Figure 4 are known to be conducted by the gut microbiota. The human microbiome can produce metabolites that modulate insulin signaling. T2DM patients have increased circulating concentrations of the microbially produced histidine metabolite imidazole propionate (XXVI). The microbiota of T2DM patients have an increased capacity to produce imidazole propionate, which is catalyzed by the bacterial enzyme urocanate reductase (**y** in Figure 4) [211], first characterized in the marine bacterium *Shewanella oneidensis* but also observed in various *Fusobacterium* and *Clostridium* spp. [212], common members of the gut microbiota.

Knudsen and colleagues [213] stated that imidazole propionate has been identified as a potential inducer of steatosis and hepatic inflammation, whereas the tryptophan metabolites indole and indole-3-acetic acid seemed to preserve liver integrity. They also listed a number of studies in which HFD- or cholesterol-fed rats and mice with MASLD were treated with prebiotics, probiotics, or synbiotics [11], and in 7/10 studies, a decrease in steatosis was observed. The probiotics used included *Lactobacillus*, *Bifidobacterium*, *Bacillus*, *Clostridium*, and *Enterococcus* spp. *Clostridium* spp. converts tryptophan to indole (Table 2), and this may be why that probiotic reduced steatosis.

## 4. Potential Therapies of MASLD Involving Manipulation of Microbiota

### 4.1. Initial Premise

With advances in 16S rRNA gene sequencing, intestinal bacteria have been discovered to play a key role in MASLD and are closely associated with the development of hepatic steatosis, fibrosis, and inflammation in animal models [214]. As discussed above, dysbiosis contributes to the development of MASLD by disrupting the intestinal epithelial barrier, allowing bacteria to translocate to the liver and bacterial toxins and metabolites to leak into the portal supply and reach the liver, where they trigger inflammation and promote fat accumulation [215]. As we have stated earlier, MASLD can progress to the more aggressive MASH, which is characterized by the presence of hepatic steatosis surpassing 5%, accompanied by indications of hepatocellular injury, such as hepatocyte ballooning, chronic inflammation, and advanced fibrosis. MASH may subsequently progress to cirrhosis, which increases the risk of HCC by about 2% per annum [1]. There is an obvious need to disrupt this chain of events, preferably at the source.

### 4.2. When Periodontal Disease Is the Cause of MASLD

One of the most readily accessible and manipulatable microbiomes is that found in the oral cavity. The dental infection of mice with *Porphyromonas gingivalis* exacerbated HFD-induced MASLD [216] after translocation to the liver [217]. Oral microbiome dysbiosis was found to be likely to cause MASLD in rats by the systemic release of the endotoxin LPS and proteases from *Porphyromonas gingivalis* [218,219,220]. In clinical studies, gentle mastication was able to induce the release of bacterial endotoxins into the bloodstream, especially when patients had severe periodontal disease. This finding suggests that a diseased periodontium can be a major and underestimated source of the chronic, or even permanent, release of bacterial pro-inflammatory components into the systemic circulation [221]. This phenomenon has since been termed the oral–liver axis [222].

Investigators employed a mouse model infected with the four aforementioned pathogens that were applied topically onto the oral cavity, and evaluated the efficacy of the antibacterial peptide nisin, a lantibiotic produced by *Lactococcus lactis*. 16S rRNA amplicon sequencing showed that, in the oral cavity, nisin reduced Proteobacteria and Fusobacteria, while increasing Firmicutes and Bacteroidetes from the infected state to control levels. In the small intestine, the nisin treatment caused minimal changes in Firmicutes and Bacteroidetes levels, but did not restore the relative abundance of ~2% of Patescibacteria (largely Saccharibacteria that grow as epibionts on host Actinobacteria and are generally regarded as part of the “microbial dark matter” [223]). In the liver, nisin increased Proteobacteria from 15% in disease to 40% relative abundance in treated animals, closer to the control hepatic levels of >50%. These authors also give details of changes in the principal bacterial genera and species in the three sites due to infection and after treatment with nisin [224].

Phage therapy (Section 3.1 and Section 3.2) has been successfully applied to treat refractory periapical periodontitis caused by *Enterococcus faecalis*. Phage PEf771 specifically infects and lyses pathogenic *E. faecalis* YN771 in patients with refractory periapical periodontitis. Not only was PEf771 as effective as a range of antibiotics in prolonging the life of rats with periapical periodontitis caused by *E. faecalis*, but it spared damage to peripheral organs. Antibiotic-treated rats developed hepatic abscesses, but not when treated with PEf771 bacteriophage [225]. The effect of PEf771 on apical healing increased with time [226]. Holins are bacteriophage-encoded membrane proteins that control bacterial cell lysis in the final stage of the bacteriophage reproductive cycle [227]. The PEf771 phage holin has been cloned and the protein expressed, and the crude enzyme of the phage holin exhibited a superior bacteria-inhibiting activity and a broader lysis host range compared to the parent phage PEf771 [228]. Moreover, the PEf771 cloned and expressed phage endolysin (bacteriophage enzymes that break down the bacterial cell wall from the inside [229]) was more effective against *E. faecalis* than the parental PEf771 phage [230].

According to data from the CDC and other sources, approximately 42% of adults in the United States aged 30 years or older have periodontitis, which is considered a periodontal infection, with roughly 8% experiencing severe periodontal disease [231]. It has been stated [224] that multiple experimental, clinical, and epidemiologic studies have established that bacterial periodontal disease is a cause of MASLD. Therefore, the latest therapeutic strategies involving antibacterial peptides, phages and their holins and endolysins offer new opportunities for the treatment of periodontal disease caused by antibiotic-resistant bacteria and consequently can be used in preventing secondary MASLD.

### 4.3. When the Gut Microbiota Is the Cause of MASLD

As has been stated, “regulation of the composition of gut bacteria may serve as a promising therapeutic strategy for MASLD” [232]. To develop this concept further, we first need to understand (i) how the gut and the liver communicate through the gut–liver axis, (ii) the impact of the gut on hepatic pathophysiology, (iii) which organisms are most influential in these processes, and (iv) which microbiota metabolites contribute to the development of MASLD.

#### 4.3.1. The Gut–Liver Axis Communication and MASLD

The number of gut microbes is of the order of 1 × 10^6^ times higher than the number of human cells, and the gut microbiota total weight is estimated to be ~2 kg. The gut microbiome comprises ~3,000,000 genes, which is 150 times that of the entire human genome [233]. The gut microbiota comprises >1000 species, distributed in more than 50 different phyla, specifically, Bacteroidetes, Firmicutes, Proteobacteria, Fusobacteria, Tenericutes, Actinobacteria, and Verrucomicrobia, making up to 90% of the total microbial population in humans. Among them, Bacteroidetes (Gram-negative) and Firmicutes (Gram-positive) are the most dominant phyla [11].

Much has recently been written on the gut–liver axis in relation to MASLD [234,235,236,237,238,239], including the gut–liver–adipose axis [240] and the gut–liver–brain axis [241]. Perturbation of the gut–liver axis occurs due to genetic background, infections, inflammation, diet (high sugar, low fiber), xenobiotics (antibiotics, drugs, food additives), and hygiene, leading to dysbiosis [242], which is an imbalance of the eubiosis within the community of intestinal microorganisms. Healthy eubiotic microflora perform a number of functions for the host, including colonization resistance to exogenous bacteria [243]; through the mucosal surface of the intestine, the microbiota interact with the host immune system, providing the host with immune regulatory functions [244], and various metabolic functions, such as breaking down complex carbohydrates and generating short-chain fatty acids, from which the host benefits [244].

When the homeostasis of the microbiota is disturbed, dysbiosis occurs in one of three ways—loss of beneficial bacteria, overgrowth of potentially pathogenic bacteria, and loss of overall bacterial diversity. In most cases, these three phenomena occur together [244]. Dysbiosis can lead to increased permeability of the intestinal mucosal barrier [245] and, in combination with compromised immunity [245,246,247], leads to the leakage into the portal vein of bacteria, bacterial products such as endotoxins, and metabolites.

#### 4.3.2. The Impact of the Gut Microbiota on Hepatic Pathophysiology of MASLD

The impact of the gut on the liver is considerable. The liver receives ~75% of its blood supply from the hepatic portal vein, which drains blood from the stomach, small and large intestines (except the distal rectum), spleen, pancreas, and gallbladder [248,249]. Bacterial products and endotoxins, such as LPS, are collectively known as pathogen-associated molecular patterns (PAMPs), which are molecular motifs associated with pathogens such as bacteria and viruses that provide signatures that are recognized by pattern recognition receptors (PRRs), not only on cells from the immune system but also epithelial cells in various tissues. PRRs not only recognize motifs associated with pathogenic microbes, but are also able to monitor the microbiota, as well as responding to motifs associated with tissue damage, known as danger-associated molecular patterns (DAMPs) [250]. Once delivered to the liver, PAMPs activate toll-like receptors (TLR) on the surface of resident liver monocytes and macrophages resulting in the increased release of various proinflammatory cytokines and chemokines, leading to hepatocyte injury and inflammatory cell infiltration. Moreover, MASLD patients had increased proinflammatory cytokine responses following exposure to PAMPs relative to healthy control subjects [251]. In addition, studies have demonstrated that MASLD patients have augmented gut mucosal permeability resulting in the increased entry of PAMPs, such as LPS and free fatty acids, into the portal circulation [252,253]. Presumably, chronic dysbiosis helps maintain the chronic nature of MASLD.

#### 4.3.3. Individual Gut Microbiota Species That Contribute to MASLD

The total number of bacterial species comprising the gut microbiota has been subject to multiple estimates. More than a decade ago, the number of culturable members of the microbiota was first estimated to be 1000 [66]. Up until today, the composition of the gut microbiota and its metabolic capacity continues to be updated [254]. In eubiosis, *Bacteroides vulgatus* and *Bacteroides dorei* preserve the intestinal wall’s integrity by upregulating tight junction expression and reducing LPS production [255]. An obesity-related translocation of gut bacteria to the liver and adipose tissues in MASLD has recently been reported. Specifically, *Enterococcus*, *Granulicatella*, and Morganellaceae abundances were positively correlated with immune cell counts and inflammatory gene expression levels. *Brevibacterium* was enriched in the adipose tissues of patients who developed liver fibrosis [256]. T2DM is an important risk factor for MASLD [1]. Evidence from experimental and clinical studies points to a reduction in butyrate-producing bacteria, such as *Faecalibacterium prausnitzii* and *Roseburia intestinalis*, as one of the most important microbiota-related features responsible for the onset and development of T2DM. Furthermore, an increase in pathogenic bacteria, such as Enterobacteriaceae, various Clostridiales, *E. coli*, *Bacteroides caccae*, and *Lactobacillus* spp., as well as *Prevotella copri* and *Bacteroides vulgates*, has been reported in the microbiota of diabetic patients [255,257].

The translocation of bacteria from the gut to the mesenteric lymph nodes is regarded as a physiological process in healthy conditions that is crucial for host immunity [258]. As stated above (Section 2.3 and Section 2.4), Proteobacterium was the most common bacterial phylum detected in blood [38,68]. Liver samples of non-morbidly obese MASLD patients contained a majority of Proteobacteria DNA, while in those of morbidly obese MASLD patients, liver histology was associated with Proteobacteria DNA as well as with bacterial DNA derived from other taxa, including Verrucomicrobiae, Actinobacteria, Nitrospira, and Bacterioidia [72].

Proteobacteria are therefore the most common bacteria found to translocate to the liver with involvement in the pathophysiology of MASLD.

#### 4.3.4. Microbiota Metabolites That Contribute to MASLD

##### Ethanol

*Klebsiella pneumoniae*, *Limosilactobacillus fermentum*, *Lactococcus lactis*, and *Enterocloster bolteae* are the main bacteria synthesizing ethanol in the microbiota, and are enriched in patients with MASH [259,260]. MASH patients had significantly higher ethanol blood levels than non-NASH obese patients and healthy controls [261]. Furthermore, MASLD and MASH patients had elevated fecal ethanol concentrations compared to controls, which were equal to their fecal levels of Proteobacteria and *K. pneumoniae*. Additionally, the high alcohol-producing strain HiAlc *K. pneumoniae* was found in 60% of patients with MASLD in a Chinese cohort [262]. These authors concluded that the microbiota drive MASLD via endogenous alcohol production.

##### Secondary Bile Acids

This subject has been extensively discussed in Section 3.3.1 above. Gut microbiome species of the genus *Clostridium*, including *C. scindens*, *C. hiranonis*, *C. hylemonae*, and *C. sordelli,* are capable of producing secondary bile acids [163]. It has been reported that secondary bile acids may be involved in MASLD progression in patients with inflammatory bowel disease, specifically ileitis [170]. A large meta-analysis of studies involving 43,229 MASLD patients and 111,578 healthy controls compared bile acids in the two groups. While 9 of 15 bile acids were increased in MASLD, conjugated and unconjugated secondary bile acids were not [174].

##### Tryptophan Metabolites

This topic has been extensively discussed in Section 3.3.2 above, where it was concluded that indole-3-propionic acid (V in Figure 3), indole-3-acetaldehyde (VIII), tryptamine (IX), and indole (X) were all end-products of gut microbiota metabolic pathways. Recent studies have established that *Bifidobacterium bifidum* metabolizes tryptophan to produce indole-3-acetic acid (VII), the precursor of indole-3-acetaldehyde (VIII). *B. bifidum* effectively prevents hepatic steatosis and inflammation through the production of VII [199]. Indole-3-acetic acid prevents steatosis by modulating lipid metabolism in the liver, primarily by reducing lipogenesis while promoting fat breakdown, achieved through mechanisms involving the activation of the aryl hydrocarbon receptor (AhR), which then downregulates key genes involved in fat synthesis, leading to decreased inflammation and oxidative stress within the liver tissue, thereby mitigating the progression of MASLD. Moreover, indole-3-acetic acid reduced hepatic stellate cell activation and pro-fibrogenic gene expression [263]. *B. bifidum* in particular is a good candidate for the probiotic therapy of MASLD.

In addition, indole (X) also activates AhR and can alleviate hepatitis and steatosis associated with MASLD [199]. As Table 2 shows, indole is produced by many gut microbiota species, namely, *Clostridium* spp., *Bacteroides* spp., *E. coli*, *Paracolobactrum coliforme*, *Proteus vulgaris*, and *Cutibacterium acnes*.

##### Histidine Metabolites

This was dealt with extensively in Section 3.3.3 above. All the catabolic pathways in Figure 4 can be carried out in the gut microbiota, with the bacteria *Klebsiella aerogenes*, *Fusobacterium varium*, *Clostridium perfringens*, *Limosilactobacillus reuteri*, and *Morganella morganii* being predominant [210]. Imidazole propionate (XXVI in Figure 4) appears to be a contributor to obesity, T2DM and MASLD, while the tryptophan metabolites (above) may alleviate MASLD. The two essential amino acids, tryptophan and histidine, with the participation of gut microbiota metabolism, seem to have opposing effects on MASLD.

### 4.4. Novel Treatment Possibilities for MASLD Involving the Microbiota

#### 4.4.1. Fecal Microbiota Transplantation (FMT)

Dysbiosis has been associated with a variety of metabolic diseases, including *Clostridioides difficile* (reclassified from *Clostridium difficile* in 2016 [264]) infections, obesity, metabolic syndrome, and T2DM. FMT is a procedure that delivers healthy human donor stool to another individual through the gastrointestinal tract, aiming to restore gut microbiota balance. FMT is believed to date back 1700 years to the Chinese alchemist Ge Hong, who first used what he called “yellow soup” to treat patients with severe diarrhea [11]. Taxonomic and functional variations in the composition of FMT have been used to treat chronic diarrhea associated with *Clostridioides difficile* infection [265,266,267]. The role of gut microbiota dysbiosis in both obesity and T2DM pathophysiology has laid the foundations for novel treatments for metabolic diseases by reshaping the gut microbiota [268]. MASLD patients have shown an increased intestinal permeability, promoting endotoxemia, increased numbers of γ-Proteobacteria, and decreased numbers of Bacteroidetes [269]. Two clinical trials of FMT in MASLD can be cited. In the first, 21 obese MASLD patients were treated with FMT from a lean vegan donor three times a week at 8-week intervals and increases in fecal microbiota abundance following FMT were seen in bacteria related to *Ruminococcus*, *Eubacterium hallii*, *Faecalibacterium*, and *Prevotella copri*. Significant changes were observed in the expression of hepatic genes involved in inflammation and lipid metabolism following allogenic FMT [270]. In the second, FMT from a healthy lean donor was administered to 21 MASLD patients and delivered endoscopically into the distal duodenum. FMT did not reduce insulin resistance or hepatic steatosis, but did have the potential to reduce small intestinal permeability [271]. While there have been some promising signs regarding the application of FMT in MASLD, this therapy awaits further refinement.

#### 4.4.2. Probiotics, Prebiotics and Synbiotics

In the early 1900s, the Nobel Laureate Ilya Mechnikov believed that the bacteria found in the colon might release toxins that ultimately lead to premature aging. He proposed changing the colonic flora for a more host-friendly flora that might promote health and longevity [272]. These ideas were the genesis of what is now probiotic therapy, and ideas of how the gut bacteria might influence disease [273,274]. Probiotics are live microorganisms that are said to improve health by restoring the balance of good bacteria in the gut. The seven core genera of microbial organisms most often used in probiotic products are *Lactobacillus*, *Bifidobacterium*, *Saccharomyces*, *Streptococcus*, *Enterococcus*, *Escherichia*, and *Bacillus*. Prebiotics have been defined by an expert panel as “a substrate that is selectively utilized by host microorganisms conferring a health benefit” [275]. Among prebiotics, only bifidogenic, non-digestible oligosaccharides, particularly inulin, its hydrolysis product oligofructose, and galacto-oligosaccharides, fulfil all the criteria for prebiotic classification [276]. Synbiotics are probiotics combined with prebiotics [277].

The use of probiotics to treat obesity in clinical trials has produced variable results [278,279,280,281]. The results of a meta-analysis of 22 randomized clinical trials of the probiotic treatment of T2DM demonstrated a significant improvement in the homeostatic model assessment of insulin resistance (HOMA-IR) following the probiotic intervention and considerably decreased glycated hemoglobin HbA1c levels and fasting blood glucose levels in T2DM patients compared to placebo. The authors concluded, “probiotics offer a promising therapeutic approach for T2DM management and warrant consideration as a potential adjunct therapy in clinical practice” [282]. Given the strong predisposition to MASLD of both obesity and T2DM, it would seem logical to evaluate probiotics for the treatment of MASLD.

In a murine model of MASLD, the administration of *Phocaeicola vulgatus* (formerly *Bacteroides vulgatus* [283]) produced the bacterial metabolite 3-hydroxyphenylacetic acid, which downregulated histone acetylation and the transcription of squalene epoxidase, a rate-limiting enzyme in steroid biosynthesis that promotes lipid accumulation in liver cells, leading to the alleviation of MASLD [284]. Regarding prebiotics, oat beta-glucan (a type of soluble dietary fiber) was administered in drinking water to mice on a western standard diet or normal chow, which induced MASLD in the former. Oat beta-glucan dampened MASLD-related inflammation, which was associated with significantly reduced monocyte-derived macrophage infiltration and fibroinflammatory gene expression, as well as strongly reduced fibrosis development. Oat beta-glucan partially reversed unfavorable changes in gut microbiota, resulting in an expansion of protective taxa, including *Ruminococcus* and *Lactobacillus* [285]. Regarding synbiotics, MASLD patients were divided into an intervention group treated with a synbiotic comprising 64 × 10^9^ CFU of *Lactobacillus* and *Bifidobacterium* (probiotic) and 6.4 g of inulin (prebiotic), and a control group treated with a placebo. Using synbiotics for 12 weeks significantly decreased liver steatosis. Synbiotics enriched the microbiomes of patients in the intervention groups with the genera *Lactobacillus*, *Bifidobacterium*, *Faecalibacterium*, and *Streptococcus* by 81%, 55%, 51%, and 40%, respectively [286]. A meta-analysis of randomized clinical trials of MASLD and obesity in children revealed that interventions involving probiotics had a positive effect [287].

#### 4.4.3. Precision Nutrition

It is clear from the foregoing (tryptophan metabolites) that certain microbiota metabolites of tryptophan, in particular, indole-3-acetic acid and indole, can alleviate MASLD. In contrast, metabolites of histidine (histidine metabolites), in particular, imidazole propionate, may predispose one to obesity, T2DM and MASLD. All the foods that are rich in tryptophan, such as chicken breast, turkey, tuna, salmon, milk, eggs, pumpkin seeds, and sesame seeds, are also rich in histidine. A study of male meat-eaters, fish-eaters, vegetarians, and vegans (98 of each) showed that both tryptophan and histidine daily intakes were of the order meat-eaters > fish-eaters > vegetarians > vegans. Despite this, these intakes were not represented by plasma concentrations of these two amino acids, with vegetarians top for tryptophan and fish-eaters top for histidine [288]. Therefore, it is difficult to design a diet that favors tryptophan over histidine. However, there are many tryptophan dietary supplements (food additives), usually in the form of 500 mg capsules. Tryptophan supplements have been associated with the sometimes fatal adverse reaction of eosinophilia-myalgia syndrome (EMS), which was recognized to be caused by impurities in batches from a particular manufacturer in Japan. After the first EMS outbreak in New Mexico in 1989, the FDA recalled all samples of tryptophan supplements. Extensive research identified more than 60 minor contaminants, of which 6 in the batches from Showa Denko KK were associated with EMS [289]. These were termed PAA, EBT, IMT, PIC, HIT, and AAA, and research also identified the stages in the industrial synthesis of tryptophan at which these impurities arose, as well as how [290]. AAA, a mixture of two structural isomers, was the only impurity statistically associated with EMS [289,290]. Since the FDA allowed the sale once again of tryptophan supplements in 2005, there have been no further cases of EMS. The treatment of MASLD with a tryptophan supplement plus a probiotic containing bacteria able to convert tryptophan to indole-3-acetic acid and indole (see Table 2) may represent a novel treatment modality for MASLD.

#### 4.4.4. Intermittent Fasting

Gonzalez and colleagues demonstrated that every-other-day fasting (EODF) in mice selectively stimulated beige fat development within white adipose tissue, and markedly improved obesity, insulin resistance, and hepatic steatosis. EODF treatment brought about modifications of the gut microbiota composition that led to the increased production of the fermentation products acetate and lactate, and to upregulation of monocarboxylate transporter 1 expression in beige cells [291]. Intermittent fasting (IF) was therefore seen as a novel means of treating metabolic diseases, including MASLD. The metabolic effects of IF have been reviewed in detail [292]. A study of Ramadan fasting, comprising 17 h of fasting/day during a 29-day period, involved 16S rRNA qPCR assays performed for the quantitation of fecal *Akkermansia muciniphila*, *Faecalibacterium prausnitzii*, *Bifidobacterium* spp., *Lactobacillus* spp., *Bacteroides fragilis* group, and *Enterobacteriaceae*. A significantly increased abundance of *A. muciniphila* and *B. fragilis* was observed in all nine subjects studied after Islamic fasting. *A. muciniphila* has been reported to be a promising therapeutic probiotic active against metabolic disorders such as obesity and T2DM [293]. The fasting response is biochemically complex, and has been reviewed, after which it was proposed that IF may offer a future method of treatment of MASLD [294].

The gut microbiota generates rhythmic oscillations in composition and function due to the drive of the endogenous circadian clock and external cues such as feeding behavior. IF alleviated obesity and MASH in mice, while reinstating rhythmicity of genera such as *Lactobacillus*, *Mucispirillum*, *Acetatifactor*, and *Lachnoclostridium* [295]. In further experiments in mice, IF influenced the diversity of the microbiota, the most significant being the increase in the abundance of *Lactobacillus* and *Verrucomicrobiaceae* represented by *A. muciniphila* [295], one of the organisms increased in the stool after Islamic fasting (see above). Regarding patients, non-pharmacological approaches to the treatment of MASLD have recently been proposed [296].

#### 4.4.5. Phages, Holins, and Endolysins

Bacteriophages or phages are viruses that infect bacteria (see Section 3.2 above). Bacteriophage lysis involves at least two fundamentally different strategies. Most phages feature at least two proteins, one of which is a hydrolase, or endolysin [297], and the other is a membrane protein, known as a holin [298]. The function of the holin is to create a lesion in the cytoplasmic membrane through which the endolysin passes. Although phage therapy has been approved by the US Department of Agriculture/Food and Drug Administration (FDA) for use in animals and plants, the FDA has not yet approved any bacteriophage products for human clinical use. However, in February 2020, the FDA recognized endolysin innovations as antibacterial biological drugs by recognizing the ContraFect endolysin in the phase III study as a “breakthrough therapy”. The pre-clinical development of additional endolysins is now underway in both industry and university labs [299]. Future treatments of MASLD may involve modifying the gut and liver microbiota to remove the offending microorganisms using bacteriophages, or their holins or endolysins, as therapeutic agents.

#### 4.4.6. Summary of Novel Treatment Possibilities for MASLD Involving the Microbiota

Fecal microbiota transplantation, probiotics, prebiotics, synbiotics, tryptophan dietary supplements, intermittent fasting, and phages and their products all represent possible treatments for MASLD. The FDA approved the use of a bacteriophage preparation made from six individually purified phages on ready-to-eat meat and poultry products as an antimicrobial agent against *Listeria monocytogenes* [300], and phages were approved by the EPA for use as pesticides on crops, such as tomatoes, citrus fruits, apples, and pears [301]. Nevertheless, there is no FDA-approved phage therapy for humans as of yet, although there are many clinical trials that have been approved by the FDA [302].

## Figures and Tables

**Table 1:** Putative atopobiotic bacteria.

Species ^‡^	Tissue	Phylum or Class *	Genus Species	Reference
Human	Urine	Firmicutes Bacteroidetes Actinomycetota	*Lactobacillus* spp. *Prevotella* spp. *Gardnerella* spp.	[32]
Mouse	Urine	Gammaproteobacteria *	*Escherichia coli*	[21]
Human	Blood	Proteobacteria * (80–90%)	*Ralstonia* spp.	[36]
Human	Blood	Proteobacteria * (>80%) Actinomycetota Firmicutes Bacteroidetes		[38]
Human	Neutrophils	Bacillota	*Staphylococcus aureus*	[41]
Human	Macrophages	Gammaproteobacteria * Bacillota Chlamydiota	*Salmonella typhimurium* *Listeria monocytogenes* *Chlamydia trachomatis*	[42]
Hamster (infected with *Opisthorchis viverrini*)	Bile	Cyanobacteria Deinococcota	*Deinococcus* spp.	[57]
Hamster (infected with *Opisthorchis viverrini*)	Liver	Fusobacteriota Bacillota Gammaproteobacteria * Actinomycetota Campylobacteria	*Fusobacterium* spp. *Streptococcus luteciae* *Escherichia coli* *Bifidobacterium* spp. *Helicobacter pylori*	[58]
Human (gallstones)	Bile	Synergistota Actinomycetota	*Pyramidobacter piscolens* *Cellolosimicrobium cellulans*	[59]
Human (infected with *Opisthorchis felineus*)	Bile	Bacillota Bacillota Bacillota Bacillota Bacteroidota Gammaproteobacteria * Alphaproteobacteria *	*Ruminococcus* spp. *Oscillospira* spp. *Anaerostipes* spp. *Dorea* spp. *Parabacteroides* spp. *Aggregatibacter* spp. *Mycoplana* spp.	[60]
White-footed mouse (infected with *Bartonella vinsonii* and *Borelia burgdorfori*)	Liver	Bacillota	*Lactobacillus* spp.	[61]
Human	Liver	Gammaproteobacteria * Gammaproteobacteria *	*Pantoea agglomerans* *Escherichia coli*	[62]
Human (HCV-related liver fibrosis)	Blood	Proteobacteria Alphaproteobacteria *		[70]
Human	Liver	Bacteroidetes		[65]
Human (morbidly obese with MASLD)	Liver	Bacteroidetes Firmicutes		[72]
Human (non-morbidly obese with MASLD)	Liver	Gammaproteobacteria * Alphaproteobacteria * Deinococcota		[72]
Mouse	Liver	Bacillota Bacillota	*Lactobacillus reuteri* *Enterococcus gallinarum*	[75]
Human	Liver	Bacillota	*Enterococcus gallinarum*	[78]
Mouse	Mesenteric lymph nodes	Gammaproteobacteria * Gammaproteobacteria * Bacillota	*Klebsiella pneumoniae* *Proteus mirabilis* *Enterococcus gallinarum*	[79]
Human	Liver	Gammaproteobacteria * Gammaproteobacteria * Actinomycetota Bacteroidota Bacillota	*Enterobacter* spp. *Pseudoalteromonas* spp. *Lawsonella* spp. *Prevotella 9* spp. *Staphylococcus* spp.	[80]

^‡^ Healthy unless otherwise stated; * Phylum or class.

**Table 2:** Gut microbiota metabolites of tryptophan.

Tryptophan Metabolite	Bacteria	Ref.
Tryptamine (IX)	*Clostridium* spp., *Ruminococcus* spp., *Blautia* spp., *Lactobacillus* spp.	[179]
Indole (X), Indole-3-aldehyde (VIII), Indole-3-acetic acid (VII), Indole-3-propionic acid (V)	*E. coli*, *Clostridium* spp., *Bacteroides* spp.	[180,181,182,183]
Indole-3-lactic acid (III)	*Lactobacillus reuteri*, *Bifidobacterium* spp.	[184,185]
Indole-3-acetic acid (VII)	*Bacteroides fragilis*, *Bacteroides thetaiotamicron*, *Citrobacter* spp.	[186]
Indole (X)	*E. coli*, *Paracolobactrum coliforme*, *Proteus vulgaris*, *Bacteroides* spp., *Cutibacterium acnes*	[187,188]

Ref. = Reference; Roman numerals refer to Figure 3.
